# Helen Rees: learning from past mistakes

**DOI:** 10.2471/BLT.21.030521

**Published:** 2021-05-01

## Abstract

Helen Rees talks to Gary Humphreys about lessons learnt in responding to epidemics, the need to maintain focus on polio eradication, and the prospects for building back better.

Q: You worked as a clinician in a Johannesburg township in the 1980s where you were also an anti-apartheid and women’s health activist. How did you get there from Cambridge University?

A: (laughing) I blame my husband who is from South Africa and left the country because of apartheid. I met him when we were both doing internships in London. After working in Zimbabwe, we returned to South Africa in the 1980s as a mixed-race couple with a one-year-old child and worked as clinicians and anti-apartheid activists in Johannesburg’s townships.

Q: You run the Wits Reproductive Health and HIV Institute, support the national government and WHO, and chair South Africa’s drug regulatory authority. How do you find time to meet all your obligations?

A: I wouldn’t be able to do it without the incredible team that leads Wits RHI and the support of my assistant. But it’s true, I am very busy and with the challenges posed by the pandemic, including the emergence of SARS-CoV-2 variants of concern, never more so.

Q: How has the emergence of the 501Y.V2 variant in South Africa affected the government’s response?

A: Plans to roll out the AstraZeneca vaccine were put on hold in February after data emerged raising concerns about the vaccine’s efficacy against mild to moderate disease caused by the variant. The government is supporting a large 3B clinical trial (a trial used to accumulate additional clinical findings) of the Johnson & Johnson Ad26 vaccine targeting health-care workers. This vaccine will soon be rolled out in groups prioritized within the general population, as will the Pfizer mRNA vaccine.

Q: How important is it going to be for vaccine makers to update COVID-19 vaccines as new variants emerge?

A: Very, particularly as these variants of concern spread. The 501Y.V2 variant – which is the dominant variant in South Africa and in many neighbouring African countries – has now been identified in over 45 countries and new variants are likely to emerge. So, we may be moving towards a scenario similar to seasonal influenza in which COVID-19 vaccines will have to be continuously modified to keep up with changing variants.

Q: What are the implications for clinical trial design and regulatory arrangements?

A: Both pre-clinical and clinical research has had to become more nimble. Classical, randomized placebo-controlled studies will be increasingly difficult to undertake, so new robust clinical trial designs will be required. It’s also vital that safety and efficacy data be generated from post-roll-out studies, and that clinical studies be developed and carried out in low- and middle-income countries. Regulators will also have to continue to be flexible in response to the pandemic, making greater use of regulatory reliance, rather than starting from scratch when they review applications. Going forward, we need to learn the lessons of our recent experiences and rethink our approach to R&D for all vaccines, and for manufacturing and distribution so that we are better prepared when the next major outbreak occurs.

Q: Which lessons do you have in mind?

A: For example, the 2014–2016 Ebola outbreak in West Africa taught us many things. First, the need for speed when outbreaks involving novel pathogens occur. In the case of the Ebola response, there were significant delays in vaccine trial implementation that were caused by the lack of pre-planning of clinical trial protocols, the lack of rapidly available funding, and the lack of earlier investment in vaccine candidates and vaccine platforms. The establishment of CEPI (Coalition for Epidemic Preparedness Innovations) has been a major step forward, notably in regard to its support for pluripotent vaccine platforms and the development of vaccines for prioritized pathogens. These were both of vital importance at the beginning of the pandemic, and have made CEPI a leader in COVID-19 vaccine R&D. We should learn from such innovations, and where possible apply new approaches to the development of other vaccines, including for example HIV vaccines where we still have yet to make a breakthrough, despite massive investment.

Q: Is enough being done to ensure that new vaccine technologies are being shared?

A: I would say not, and without changes to intellectual property protections this is not likely to change. Notwithstanding the obvious sensitivities on this topic, I think there is an argument for the temporary suspension of intellectual property rights during PHEICs (Public Health Emergencies of International Concern). The TRIPS (Trade-Related Aspects of Intellectual Property Rights) flexibilities come close to this in regard to “national emergencies” and “other circumstances of extreme urgency” where there is no need for a government to try first for a voluntary licence from the patent owner. I think efforts should be made to ensure that countries avail themselves of this flexibility, perhaps with a specific link to PHEICs.

Q: Do you see any potential for a patent pool for COVID-19 vaccines like the Medicines Patent Pool?

A: The COVID-19 Technology Access Pool created by WHO last year was supported by low- and middle-income countries and a few wealthier ones, but industry did not generally get behind it. Of course, patents are not the only issue here. There is also the question of capacity. The pandemic has been a wakeup call with regard to the lack of manufacturing capacity in many regions and efforts should be made to ramp up capacity worldwide. The African Union is prioritizing vaccine manufacturing and regional procurement which are both initiatives that could expand beyond COVID-19 vaccines. In addition, CEPI is investing in new vaccine manufacturing technologies that have the potential for application in low-resource settings.

Q: The focus on COVID-19 has caused other immunization programmes to be neglected. Which are of most concern to you?

A: There are three issues of great concern. First is the impact of the pandemic on routine immunization coverage. Limited access to services and fear of COVID transmission in facilities has resulted in falling immunization rates. WHO and other partners have been working closely with countries to reverse this trend and to ensure readiness for the provision of COVID-19 vaccines. Second, the recurrent outbreaks of Ebola in the DRC (Democratic Republic of the Congo) suggest that Ebola outbreaks are likely to continue for the foreseeable future. So it is vital that we remain vigilant, making full use of the ring vaccination strategies which have been successful thus far. Third, as chair of the IHR (International Health Regulations) Emergency Review Committee on polio, I’m very conscious of where we stand with regard to global polio eradication efforts, and am concerned about the spread of circulating vaccine-derived poliovirus (cVDPV).

“The national stockpiling of vaccines makes no epidemiological sense and is morally unacceptable.”

Q: Can you explain in simple terms what cVDPV is?

A: Oral polio vaccine (OPV), the most commonly used polio vaccine in low- and middle-income countries, contains a weakened form of the poliovirus, which triggers the desired immune response in the body. The attenuated virus replicates in the intestine for a limited period and can be excreted. In places where sanitation and access to clean water is lacking, the virus can be transmitted. Where the local population is under-immunized, excreted virus can circulate and evolve, with potential to change into a form that paralyses. That is what we are seeing in an increasing number of countries in the African Region and beyond.

Q: The increase of cVDPV has been linked to the decision to remove the type 2 polio component from the trivalent oral vaccine in 2016. Was that decision a mistake?

A: Hindsight is a wonderful thing, but you have to remember that the removal of the type 2 component was associated with significant public health benefits, including a reduction of the risk of cases of cVDPV2, given that until 2015, over 90% of cVDPV cases were due to the type 2 component in OPV. Also, you have to bear in mind that the transmission of wild poliovirus type 2 had been interrupted since 1999. But the bottom line is that when we dropped the type 2 component we created many susceptible populations at enormous scale. Not only that, but we missed vaccinating certain populations of children altogether. What we are also seeing is the exportation of cVDPV across borders and often in countries with weak health services and surveillance. So the spread of cVDPV is a major concern, made worse during the pandemic because of the suspension of immunization campaigns.

On the positive side, we now have the novel oral polio vaccine (nOPV2), which clinical trials have shown provides protection comparable to the type 2 vaccine, while being more genetically stable and less likely to give rise to cVDPV2 in low-immunity settings. So, I’m hopeful that the nOPV2 will help us maintain momentum towards eradication, as long as the political will is there and support for eradication is sustained.

Q: Are there any other new vaccines that have got your attention?

A: There is research going on regarding a single dose HPV (human papillomavirus) vaccine which if found to be effective would be a wonderful boost for HPV vaccine programmes and would support the ambitious goals set for cervical cancer elimination. Cancer of the cervix is still a predominant cancer in many low- and middle-income countries, particularly in the African Region where screening is challenging. We are also seeing the emergence of other HPV-related cancers in the northern hemisphere, including oral/pharyngeal cancers for example, so the need for widely available vaccines remains a global priority.

Q: There has been talk of building back better after the epidemic. What would that mean for immunization programmes?

A: Building back better would involve innovation on several fronts, including a renewed commitment to global equity for vaccine access. For COVID-19, vaccine nationalism and the national stockpiling of vaccines makes no epidemiological sense and is morally unacceptable. Either we are going to keep repeating the mistakes of the past or, as a global community, we are going to tackle some of them. My hope is that the global discussion regarding vaccine hoarding and refusal to export is the beginning of a broader debate about vaccine access and equity. I am also hopeful that the COVAX facility will drive improved access. With COVAX vaccines now reaching 100 countries, we may be witnessing the beginning of a new global health success story.

